# Commentary: Brief report - Monoclonal antibodies illustrate the difficulties in measuring blocking TSH receptor antibodies

**DOI:** 10.3389/fendo.2022.1060280

**Published:** 2022-10-25

**Authors:** Paul D. Olivo, George J. Kahaly

**Affiliations:** ^1^ Department of Molecular Microbiology and Microbial Pathogenesis, Washington University Medical School, St. Louis, MO, United States; ^2^ Molecular Thyroid Research Laboratory, Department of Medicine I, Johannes Gutenberg University (JGU) Medical Center, Mainz, Germany

**Keywords:** thyrotropin receptor, thyrotropin receptor antibodies, thyroid-stimulating antibodies, thyroid-blocking antibodies, monoclonal antibodies, bioassays

## Introduction

The authors read with interest the article entitled “Brief Report - Monoclonal Antibodies Illustrate the Difficulties in Measuring Blocking TSH Receptor Antibodies.” published in 2022 in *Frontiers in Endocrinology* (1). The experiments with mixtures of monoclonal antibodies with either stimulatory or blocking activity were interesting and provocative. The discussion of the results was also enlightening.

## Commentary

The authors would like to note that Davies et al. were not the first to perform experiments testing mixtures of thyrotropin receptor (TSH-R) monoclonal antibodies (mAb) in bioassays. In 2013 the authors published an article in *Clinical Experimental Immunology*, not cited in Davies et al., in which the authors used mixtures of the purely stimulatory mAb, M22, and the purely blocking mAb, K1-70, and measured the activity of these mixtures in bioassays for thyroid-stimulatory antibody (TSAb) and thyroid-blocking antibody (TBAb) (2). We refer to Figure 7 of our manuscript, which shows the results of testing 11 mixtures of each mAb in 10% incremental proportions from 100:0% to 0:100% using the concentration of each mAb that gave maximal stimulation (M22; 20 ng/mL) or inhibition (K1-70; 200 ng/mL). We found that we could detect TSAb activity even in the presence of up to 60% K1-70, and that we could not detect TBAb activity when the K1-70 was less than 60%. Conversely, we could not detect TSAb actvity when the K1-70 was greater than 60%, and we could only detect TBAb activity when the M22 was less than 40%. Interestingly, we observed ‘negative inhibition’ (stimulation) in the TBAb bioassay when the M22 was 40% or greater. From these results we can conclude that the actvities of both TSAb and TBAb bioassays can be affected by high levels/affinity/potency of antibodies of the opposite actvity.

In subsequent work, in assaying sera for blocking activity, a cell-based TBAb bioassay was found to be about 20 times more sensitive than a commercial TSH-R-binding assay that did not discriminate between stimulatory and blocking activity (3, 4). Furthermore, mixtures of blocking and stimulating mAbs were tested in the blocking bioassay. At 100% K1–70 (200 ng/mL), 80% K1–70 + 20% M22, 60% K1–70 + 40% M22, 40% K1–70 + 60% M22, 20% K1–70 + 80% M22, and 100% M22 (20 ng/mL), we observed 82%, 61%, 24% (negative), –26% (negative), –77% (negative), and –95% (negative) inhibition, respectively (3). Therefore, the blocking bioassay not only detected blocking antibodies, but it also detected the presence of stimulatory antibodies, reporting them as negative inhibition, although it is not as sensitive as the stimulatory bioassay (2, 3, 5).

Recently, similar observations using stimulating and blocking mAb mixtures have been made, expressed as the relationship between TSH-binding inhibitory immunoglobulin (TBII) and bioassays (6, 7). These studies showed that the characteristics of TSH-R-Ab in certain patient serum can be mimicked by mixtures of human anti-TSH-R mAbs, and suggested that these sera contained mixtures of stimulatory, blocking, and in once case, neutral anti-TSH-R-Ab.

## Discussion

The previously published results described above demonstrate that the effect of competing TSH-R-Ab activities is not just an issue for TBAb bioassays, as stated in Davies et al., it is relevant to TSAb bioassays as well. We agree, however, that it is probably more likely to be an issue with TBAb bioassays especially when testing serum from Graves’ disease (GD) patients who have high levels of TSAb. Nevertheless, we conclude that both bioassays measure the net bioactivity in these mAb mixtures and that, by extension, it is the net actvity that is measured in a serum sample. We also would propose that it is the net actvity that correlates with risk for hyper- or hypothyroidism (8). We believe this notion is consistent with the preponderence of data in the literature regarding bioassay results.

## Author contributions

All authors listed have made a substantial, direct, and intellectual contribution to the work and approved it for publication.

## Conflict of interest

PO and GK consult for Quidel Corporation San Diego, CA, USA.

## Publisher’s note

All claims expressed in this article are solely those of the authors and do not necessarily represent those of their affiliated organizations, or those of the publisher, the editors and the reviewers. Any product that may be evaluated in this article, or claim that may be made by its manufacturer, is not guaranteed or endorsed by the publisher.
